# Generation Y and surgical residency – Passing the baton or the end of the world as we know it? Results from a survey among medical students in Germany

**DOI:** 10.1371/journal.pone.0188114

**Published:** 2017-11-27

**Authors:** Robert Kleinert, Claudia Fuchs, Vanessa Romotzky, Laura Knepper, Marie-Luise Wasilewski, Wolfgang Schröder, Christiane Bruns, Christiane Woopen, Jessica Leers

**Affiliations:** 1 Department of General-, Visceral- and Cancer Surgery, University of Cologne, Cologne, Germany; 2 Department of Palliative Medicine, University of Cologne, Cologne, Germany; 3 Institute for the History of Medicine and Medical Ethics, University of Cologne, Cologne, Germany; Medizinische Universitat Graz, AUSTRIA

## Abstract

**Introduction:**

The current student generation have their own expectations toward professional life and pay particular attention to their work-life balance. Less interest in work-intensive specialties leads to a shortage of skilled candidates especially in surgery. In order to motivate students into a surgical residency, new priorities become important. A deeper understanding of the underlying arguments and students’ expectations towards a surgical training are necessary to counteract a future shortage of specialized surgeons.

**Methods:**

We conducted an internet-based survey among medical students at two representative German university hospitals to gain more information about the underlying mechanisms that lead to opting for and against a surgical career. We particularly paid attention to gender differences and differences between students of different academic years.

**Results:**

A total of 1098 students participated in the survey. Sixty-four percent were female. The majority of the students were of the opinion that surgery is an interesting and meaningful profession. In contrast, when it comes to their own career choice, most students (89% female and 81% male) are not willing to choose a surgical specialty. While students are certainly willing to spend a large amount of time on their professional lives, at the same time they demand planning reliability and a sufficient work-life balance. Flexibility in working hours and an existing childcare program were identified as predominant factors for all students and in particular for female students. The same applies to a respectful conversional tone and appreciation of the individual work. Factors like prestige and salary were less relevant than “self-fulfillment” in terms of respectful interaction and balancing their working and private lives. There was significant difference in female and male students as female students have clearer ideas concerning career planning but at the same time are less self-confident than their male colleagues. Moreover, there was a significant difference between junior and senior students regarding career planning with a shift to less work-intensive specialties and especially away from a surgical residency in older students. Adjustments to working hours models, working environment, clinical curriculum and a respectful interaction are factors that might increase the willingness of young students to choose a surgical career.

## Introduction

The surgical profession is among the most fascinating fields in medicine. However, in the western world the willingness of medical students to enter a surgical residency is decreasing [1, 2].

The surgical profession is characterized by strong personal dedication and long working hours along with often unpredictable work schedules. Ideally, the surgeon subordinates his profession to everything else and this is an idea that is still embedded in students’ heads.

Furthermore, surgical residency is different to residencies in other fields as learning of surgical procedures on top of theoretical background knowledge makes training demanding and time-consuming [2]. Practical training in the OR is characterized by a master-disciple relationship and thus potentially negatively affected by subjective interpersonal relations and strict hierarchies.

These facts are contrary to the self-perception and values of the current student generation who are known to have unique expectations toward work-life balance, learning style [3] and career hierarchies [4]. Furthermore, it collides with family planning as an increasing number of women entering the medical field [5].

Hence, the rate of students in the current generation who are willing to choose a surgical career [6, 7] is declining. When there is inter-hospital competition for skilled physicians, new priorities come to the fore [8]. They include considerations of expectations and demands of young students with respect to private life and career planning [9]. These relationships are increasingly discussed in literature [10–12]. However, most surveys focus on the description of demographic factors rather than analyzing students’ individual opinion and motivation. A deeper understanding and weighting of the underlying reasoning and students’ requirements on surgical training is mandatory to counteract a future shortage of specialized surgeons [11] and would help to recruit the most promising candidates for a surgical residency [6, 13].

Therefore, we conducted a survey among medical students at two representative German medical universities to gain more information about the underlying mechanisms that lead to opting against a surgical career and to identify possible solutions for enthusing more students to choose a surgical residency. Content of the questionnaire covered the most common prejudices about the surgical field and the demands of young students towards continuing education in the surgical field.

## Materials and methods

Between January 2015 and May 2015, a total of 1098 students participated in the survey by answering 60 questions in a web-based online survey. Beneath general questions regarding their career and family plans, we specifically asked about expectations towards future workplace and requirements on medical teachers and colleagues. Questions were categorized in thematic modules as summarized in Table 1.

**Table 1:** The survey included 60 questions that were grouped into 9 thematic modules. For some questions, additional text fields were provided to allow respondents to write out their answers freely.

Thematic modules	Number of items
Personal data	10 items
Requirement on future career	16 items
Expected obstacles in future professional life	5 items
Plans for career start	6 items
Prioritization of personal plans	4 items
10 year career goals	9 items
Opinion on surgery	12 items
Expected obstacles for a surgical residency	8 items

In order to reveal the underlying mechanisms that lead to opting for or against a surgical career we defined hypotheses on student expectations and demands. Table 2 summarizes the underlying hypotheses.

**Table 2:** Underlying hypotheses with corresponding items as a basis for questions.

Hypothesis	Items
**Low attractivity of the surgical field**	Meaningful occupation
	Social esteem
	Research opportunity
	Flexibility in career planning
	Intellectual challenge
	
**There is lack of respect**	Harsh tone
	Hierarchical working einviroment
	Lack of teamwork
	
**Unbalanced gender composition**	Leading role
	Impairs familiy life
	
**Work-life balance**	High workload
	Childcare program
	Salary is too low
	Flexible working hours model
** **	
**Education**	Job rotation
	Research opportunity
	Quality of training

Underlying items were repeated in different thematic modules as control questions. Questions were designed and grouped under supervision by a psychologist to exclude student influence caused by leading questions and consequently this ensures a standardized and valid questionnaire. The survey was conducted at two German University Hospitals (University Hospital of Cologne and University Hospital Goettingen). Students were contacted via student representatives and answered a web-based questionnaire on a voluntary basis. Survey was anonymous and answers were stored without any personal connection. Hence our Ethics committee at the University of Cologne approved the survey on October 2015 (Regulation Number 155–143) and decided that the survey no written consent is necessary as participation was on a voluntary and anonymous basis. Answers were analyzed anonymously. Personal data included information regarding decision-making in the medical specialization and whether a surgical career is planned or imaginable. The questions were either answered using a four point “forced choice” Likert scale (1 = not reasonable, 2 = partly reasonable, 3 = reasonable, 4 = very reasonable) or with “yes” or “no” answers. Answers were further divided into the categories “no” which included Likert scale items 1 and 2 and “yes” including Likert scale items 3 and 4 in order to analyze the answers using the Mann-Whitney U test. The student population was divided according to their academic year into “junior students” (up to 4 semesters) and “senior students” (5 semesters and above). This threshold was intentionally chosen as the majority of the students enter the clinical phase at the earliest at the end of their 4th semester. Up to this point in time they do not have sufficient insight into daily clinical routine which starts with the clinical phase in their 5th semester.

A p< 0.05 was considered significant. Correlation between items was analyzed using a cross-tab chi square test.

## Results

A total of 1098 medical students (equaling a return rate of 19.6%) participated in the survey. 64 percent were female and 36 percent were male. The mean age was 25 years and mean semester was the 6^th^ (Table 3).

**Table 3:** Summary of the participating students. A total of 1098 students participated in the survey. 11% of all female students and 19% of the male students are planning to choose a residency in a surgical field.

Study population	total	percent
**Total number of participants**	**1098**	**100**
* female	705	64
* male	393	36
**Mean age of participants**	25 ± 4 years	
	** **	
**Current semester**	6 ± 3 semester	
* up to 4th semester	n = 525	
* 5th semester and above	n = 573	
**Students planning a surgical specialty**	**158**	**14% of total**
* female	82	11% of all female
* male	76	19% of all male

14% of the students planned to choose a surgical career. This included all surgical residency programs (e.g. in General, Trauma, Visceral and Heart Surgery) as well as specialties which include training of “classic” surgical skills such as in Orthopedics, Gynecology or Urology. Difference between male and female students was not significant.

### Student expectations and requirements

Students were interviewed about the demands and expectations they have of their future career. All students pay particular attention to respectful working relationships as they demand a collegial conversational tone. Furthermore, they pay attention to balancing their family and careers in terms of the following parameters: “flexible working hours model”, “flexibility in career planning” and “existing childcare program” (Table 4). The items “Work-life balance” and “Quality of training” were very important demands as well as salary. Academic year, i.e. seniority, influenced the demands and expectations as more advanced students voiced significantly higher demands than younger students.

**Table 4:** Total number and percent of female and male students that answered questions regarding future expectations on their working environment.

Students expectations		considered important	considered important	significance
		male	female	academic year
	**Demands on future working environment**			
	Collegial conversational tone	n = 349 (88%)	n = 663 (94%)	significant increase
	Gender composition	n = 72 (18%)	n = 226 (32%)	significant increase
	Quality of training	n = 347 (88%)	n = 647 (92%)	significant increase
	Job rotation	n = 72 (18%)	n = 226 (32%)	significant increase
	Practical activity	n = 237 (60%)	n = 385 (55%)	significant increase
	Prestige	n = 167 (42%)	n = 168 (24%)	not significant
	Gender composition	n = 72 (18%)	n = 226 (32%)	significant increase
	Research possibility	n = 142 (36%)	n = 305 (43%)	significant decrease
	**Balancing family and career**			
	Flexible working hours model	n = 289 (73%)	n = 610 (87%)	significant increase
	Flexibility in career planning	n = 326 (83%)	n = 611 (87%)	significant increase
	Existing childcare program	n = 198 (50%)	n = 541 (76%)	significant increase
	Work-life balance	n = 326 (86%)	n = 636 (90%)	significant increase
	Salary	n = 284 (72%)	n = 473 (67%)	significant increase

In the next stage, students were asked about their feared hurdles regarding their first professional position. Senior students are more concerned about excessive professional demands made on them than younger students are. However, older students are significantly less concerned about high physical stress than younger students. Interestingly, gender composition is not a frequently used argument.

**Fig 1:**
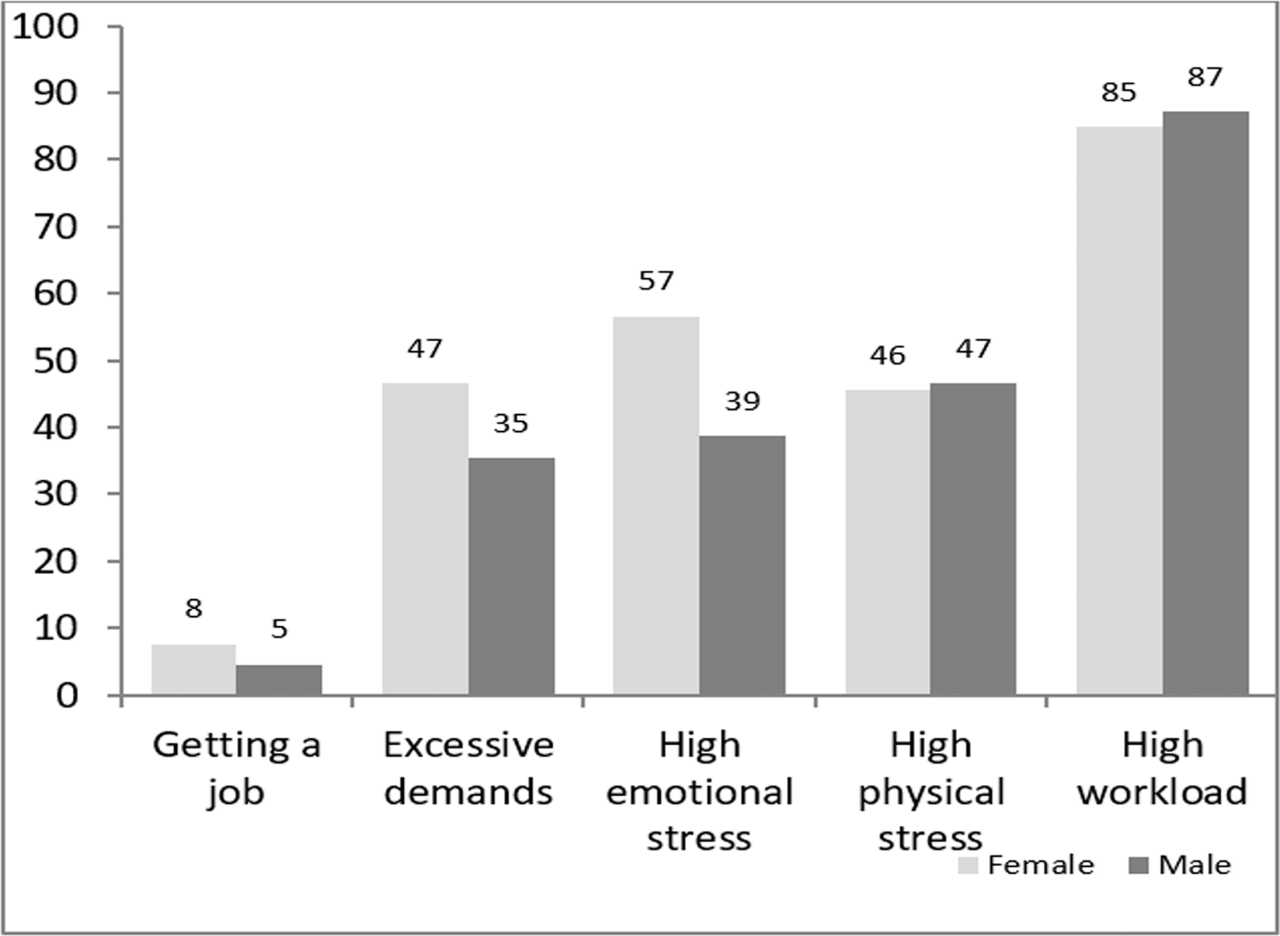
Feared hurdles regarding their first professional position expressed as a percentage of students who “agree” or “fully agree”.

The majority of students judge a high workload as a hurdle, whereas only a few students are concerned about job opportunities (Fig 1). However, many students are ready to face high emotional and physical stress and do not fear excessive demands.

**Fig 2:**
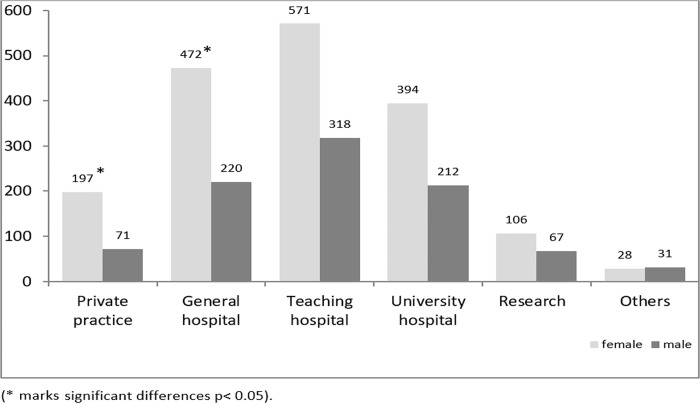
Summary of the question: “Where do your plan your career start?” percent of students who agreed.

To assess their future plans, students were asked about their career start (Fig 2).

**Fig 3:**
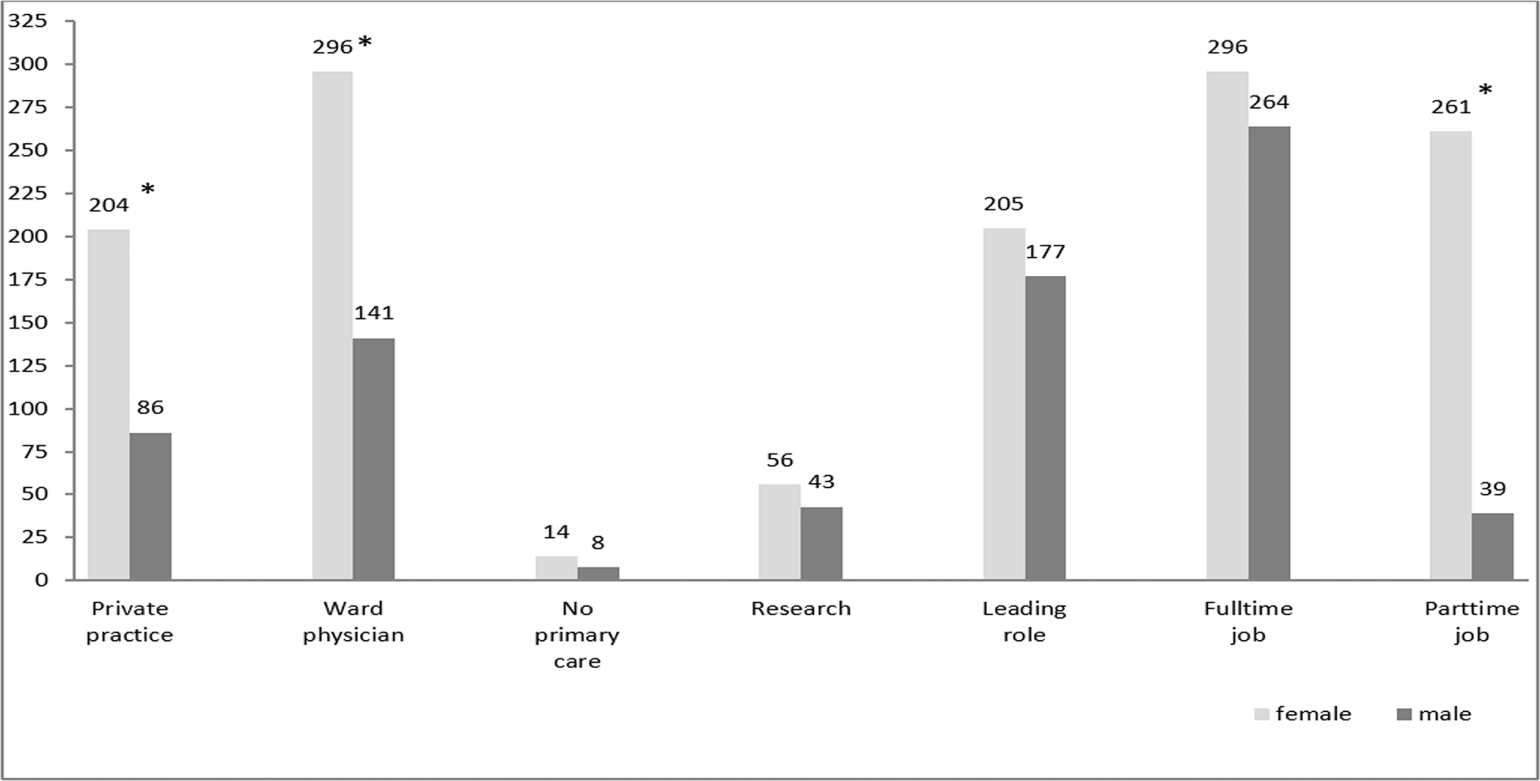
Long-term planning for all students expressed as a percentage of positive answers for male and female.

The majority of the students plan to start a career in a teaching hospital. In order to specify their future plans, students were asked where they see themselves in 10 years (Fig 3). Most of the students could imagine working as a hospital physician. Working in a private practice was less attractive. The majority of the students are willing to work full time (4).

**Fig 4:**
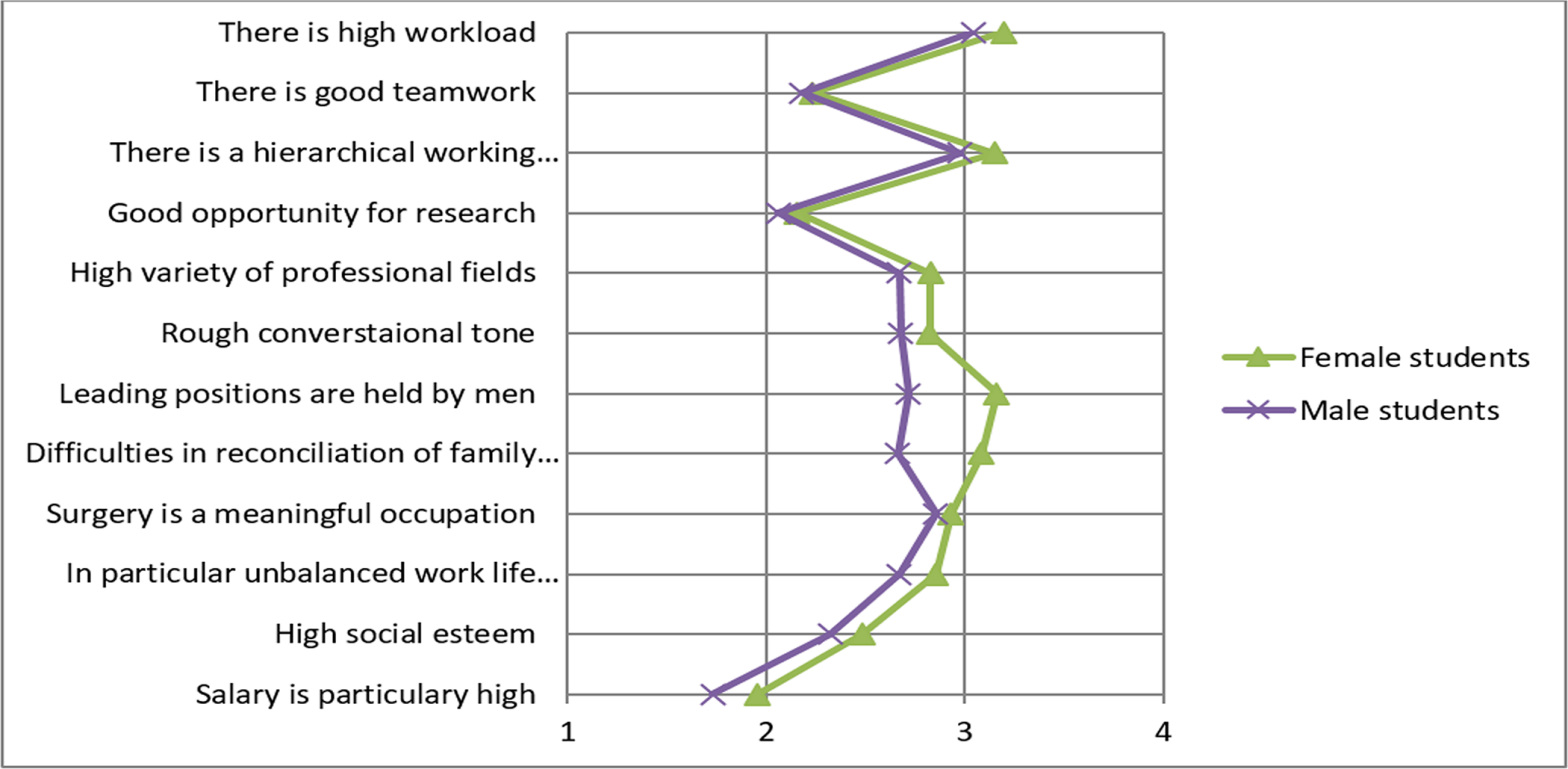
Opinion about surgery was assessed by asking 12 questions.

Finally, students were asked to rate their expectations about surgery on a scale from 1 (disagree) to 4 (agree). Students are of the opinion that working in surgery is characterized by a high workload, a hierarchical working environment and a rough conversational tone (Fig 4). However, most of the students agree that surgery is a meaningful occupation. Interestingly, most of the students disagree with the statement that working in surgery is characterized by good teamwork.

**Fig 5:**
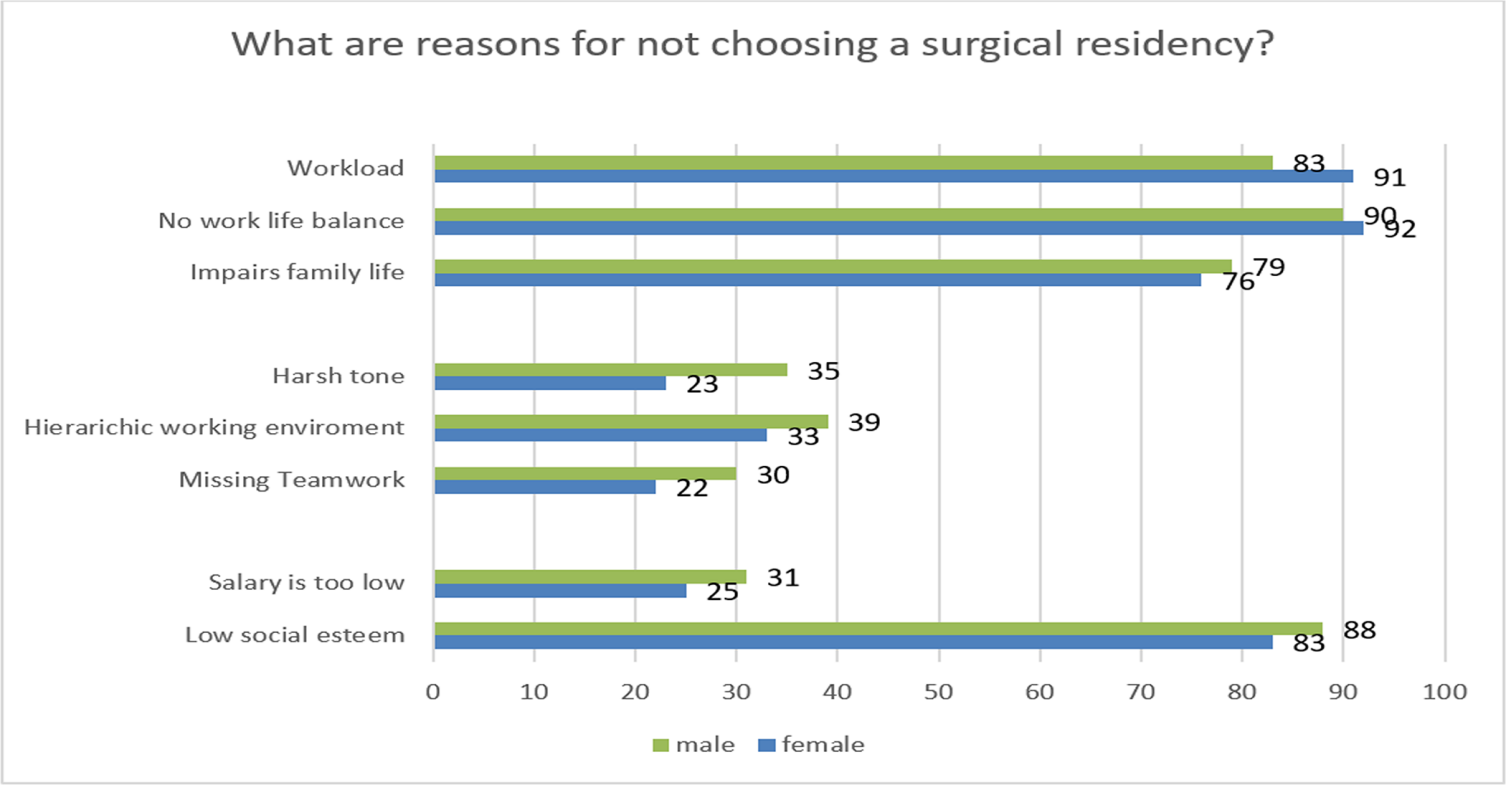
What do you see as obstacles to choosing a surgical residency?

Consequently, we asked about possible reasons for not choosing a surgical residency (Fig 5).

Only 11% of all female students plan to choose a residency in a surgical field compared to 19% of the male students. Students criticize a high workload and subsequently a low work-life balance. Interestingly, although they see surgery as involving a high workload they think that salary for surgeons is fairly sufficient. These considerations lead to the assumption that surgery in general is not interesting.

To sum up, students fear a lack of respect toward future residents and an inordinately heavy workload with an inadequate work-life balance [14]. Most students are willing to work full time and could imagine working on a ward in the long run. However, they require high-quality job training with a certain amount of flexibility.

## Discussion

This study describes the underlying mechanisms influencing the decision for or against a surgical career among generation Y. The majority of students are of the opinion that surgery is an interesting and meaningful profession. In contrast, most of the students are not willing to choose a surgical career, which is also reported by other authors in other European countries [6]. This raises the question whether clinical teachers could influence motivations for choosing a surgical residency.

For the current student generation work-life balance is an extraordinarily important factor often not negotiable and thus assumed to be superior to all other factors [15]. It is often believed that this prevents many medical students from choosing a career in primary healthcare and in particular in surgery. These findings are also supported by other authors [12]. Surgery is a profession with considerable responsibility and characterized by great personal dedication. Yet, to believe that generation Y is generally not willing to become engaged in such responsible positions would be a superficial view that fails to understand the attitude of the current student generation. This survey reveals that the current student generation is afraid of a high workload. However, the majority of the students aim for a leading full-time position. For the majority of the students polled, factors like prestige and salary are less relevant than respectful interaction and balancing work and private life.

“Work-life balance” is based on subjective perception rather than on objective definitions and can therefore be interpreted individually. As a result, we defined factors that can possibly break down “work-life balance” and identify underlying factors such as flexibility in working hours and an existing childcare program [16]. The majority of students are of the opinion that these factors, which can be summarized as “balancing one’s professional and private life”, are of the utmost importance. These expectations are incongruent to the expectations that students express when thinking about working in surgery. The majority of the students polled feel working in surgery is closely linked with difficulties in combining family and working life resulting in a particular skewed work-life balance [14]. Our results underline the opinion that adjustments to working hours models or working environment are necessary in order to motivate students to choose a surgical career [17].

The comparison of junior and senior students revealed that the tendency to opt against a surgical career is higher in older students. This is a remarkable result even though the present survey was designed as cross-sectional and not as longitudinal study.

The significant increase in the numbers opting against this career choice as they go from being a junior to a senior student suggests that subjective experiences during medical school affect career aspiration away from a surgical residency. This is underlined by the fact that most students in our survey are planning to choose a career start as resident in a full-time position. In the long run, there is a significant shift from a conceivable position as ward physician towards private practice between junior and senior students. This should lead to a critical examination of the question whether modification of the surgical curriculum could possibly counteract this trend.

Nowadays, female students form the largest group of students. Therefore it is mandatory to specifically analyze this group. Hill et al. propose the thesis that for female students, “paradigmatic trajectories” are a main factor for opting against a surgical career [18]. In our survey this factor (role models) was not identified as one of the most important requirements on the future working environment. However, female students were of the opinion that men mainly hold leading positions in surgery, which potentially is an indirect sign that supports the role model theory [11].

The current study was limited on two representative medical faculties in Germany assuming that this sample allows a conclusion concerning all medical students. The results are possibly biased as the participation was on a voluntary basis with a response rate of 19.6%. However, there are only a few surveys with similar or slightly higher number of students. Although this survey included German students, the results are potentially transferable to other countries as there are similar reports about the small proportion of students who are willing to choose a surgical career [13].

To sum up, our survey reveals that the current student generation is motivated and willing to spend a certain amount of time on their professional lives but that they have clear ideas about “self-fulfillment” and self-confident expectations on their future workplace. The responsible surgical managers should consider further enhancing clinical education, improving working environments and paying attention to respectful professional interaction. Furthermore, we should be aware that according to our data a good percentage of students negatively change their view on surgery based on their final years at medical school.
